# Supplementary material for: The Production of Three-Dimensional Metal Objects Using Oscillatory-Strain-Assisted Fine Wire Shaping and Joining
Source: Materials (Basel). 2024 May 7;17(10):2188. doi: 10.3390/ma17102188 (PMC11122744; doi:10.3390/ma17102188)
Supplement: Supplementary file 1 [file materials-17-02188-s001.zip › Supplemental material_1.pdf]

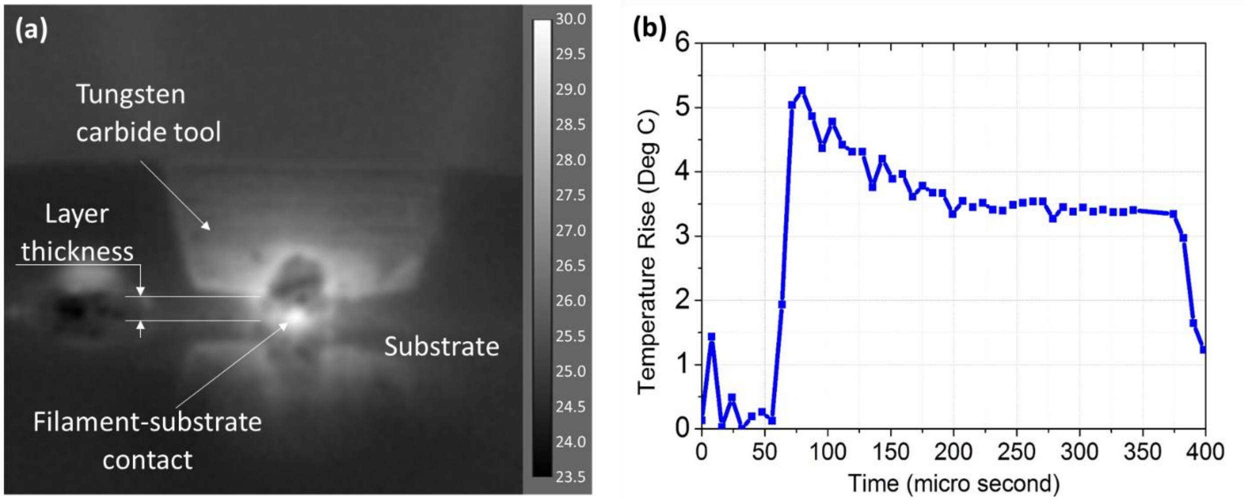

Supplemental Figure 1. (a) one frame of a thermal video of cross-section view of the tool, wire, and substrate during RAD voxel formation. It shows spatial temperature distribution at the time point where the maximum temperature is reached (at the filament-substrate contact) in the field of view. The temporal evolution of the temperature at the filament-substrate contact is shown in (b). The numerical values used to calculate temperature rise in the plot were extracted from the thermal video. The entire voxel formation time is 0.3 s. Two main heat sources in RAD are the frictional heating on the filament-voxel interface and the cyclic plastic strain coupling into heat dissipation. Frictional heat starts as the voxel formations begins. It terminates as the metallurgical bonds form at approximately 30 milli seconds into the process. The cyclic plastic strain heating goes on until the ultrasound vibration stops at the conclusion of the voxel formation [15].

Analysis of oscillatory-strain assisted enhanced diffusion

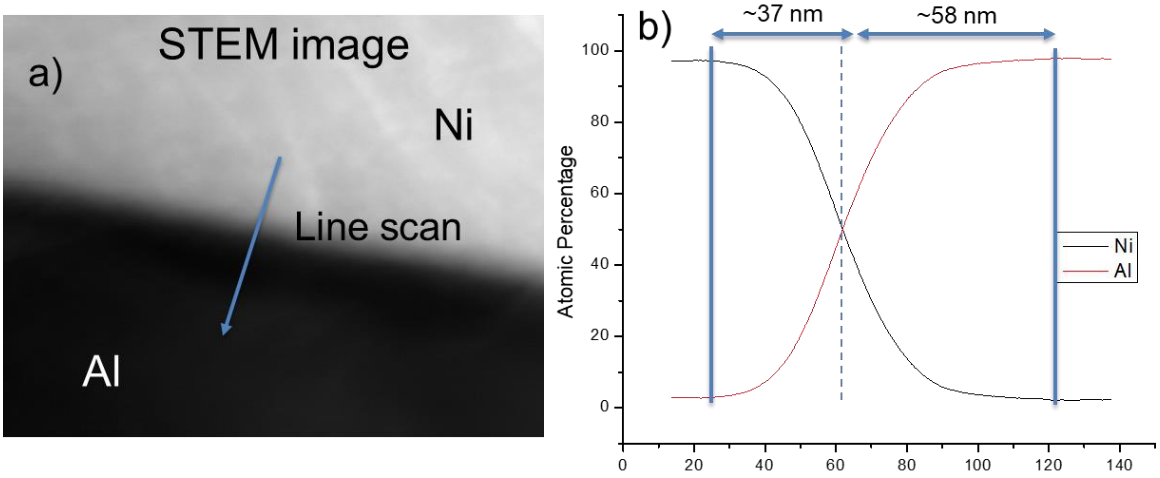

Supplemental Figure 2. Left: STEM Image of Al-Ni interface formed by using the proposed RAD technique. Right panel: EDS scan across the Al-Ni interface showing relative changes in atomic percentages across the interface.

Supplemental Figure 2 shows the STEM image of the Al-Ni interface after depositing pure Aluminum voxel on pure Nickel substrate along with the results of an EDS line scan which shows atomic percentage of each constituent versus distance after smoothing the curves. The resulting width of the inter diffusion zone was found to be ranging between ~80 nm to ~140 nm. A peculiar feature of the diffusion profile shown in **Error! Reference source not found.**b is that the width of the inter-diffusion zone is larger on the aluminum side of the interface. This feature is consistent for all the diffusion profiles. This can be attributed to the region with high defect density observed in aluminum side. Higher defect density in this

region leads to a more open aluminum lattice leading to increased diffusivity of nickel in aluminum. This increased defect density was also observed to assist in bond formation in ultrasonic welding where very high diffusivity of copper was observed in aluminum[18].

The diffusion profile shown in b was used to calculate the effective inter-diffusion coefficient of aluminum and nickel using Wagner's approach [19]. The objective of this analysis is to get an approximate value of the inter-diffusion coefficient. Hence, for simplicity, the assumption made is that the interface temperature remains constant throughout the bonding process.

According to Wagner's approach, the inter-diffusion coefficient is given by

$$\tilde{D}(Y_B^*) = \frac{v_m^*}{2t(dY_B/dx)_{x^*}}\left[(1-Y_B^*)\int_{x^{-\infty}}^{x^*}\frac{Y_B}{v_m}dx + Y_B^*\int_{x^*}^{x^{\infty}}\frac{(1-Y_B)}{v_m}dx\right]$$

where,

x is the position along the curve,

v_m is the molar volume at composition of interest,

$Y_B^* = \frac{N_B^* - N_B^-}{N_B^+ - N_B^-}$, N_B is the atomic fraction of nickel and N_B^+ and N_B^- are the nickel atomic fraction at the two extremes of the curve.

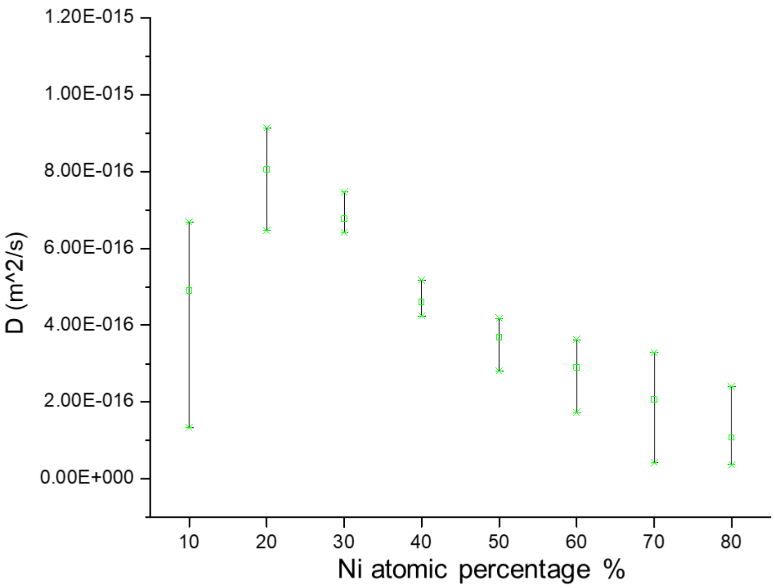

Supplemental Figure 3. Graph of inter-diffusion coefficient between aluminum and nickel as a function of Ni atomic percentage for energy density of 310 J/m³.

Supplemental Figure 3 shows the graph for of inter-diffusion coefficient between aluminum and nickel as a function of Ni atomic percentage for energy density of 310 J/m^3. The values range from ~1x10^{-16} m^2/s to ~9x10^{-16} m^2/s. By using the Arrhenius equation, $D = D_0 e^{-\frac{Q}{kT}}$ and using the values of D_0 and Q from the literature[20,21], the effective constant temperature that would be required to achieve a diffusion zone of the size obtained in the experiments was calculated. The effective temperature values range from ~320 ^{0}C to 420 ^{0}C.

These temperatures values are much higher than the values from high speed infra-red camera measurements performed during the bonding process [15].

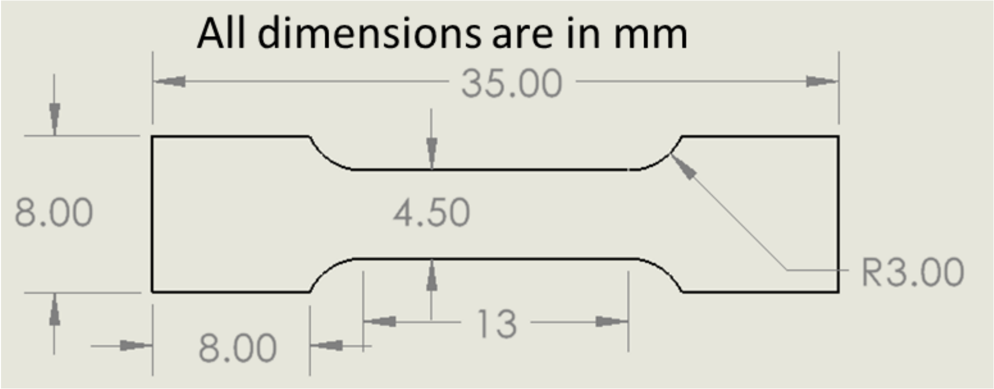

Supplemental Figure 4. Figure shows the geometry of tensile test coupons. Thickness = 4mm.

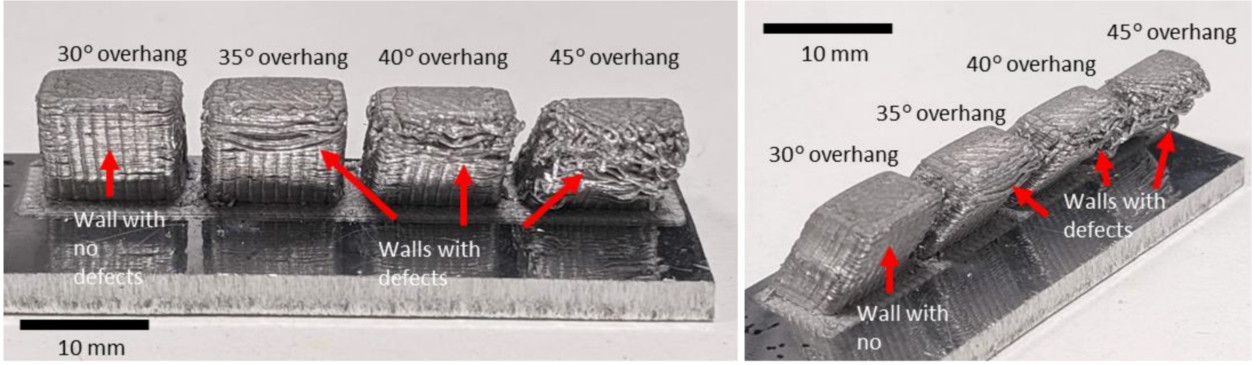

Supplemental Figure 5. A sample printed with varying degrees of overhang. The overhang angle is the angle made by the walls with the vertical. Overhangs up to 30° can be printed consistently.
